# Missing and overexpressing proteins in domestic cat oocytes following vitrification and in vitro maturation as revealed by proteomic analysis

**DOI:** 10.1186/s40659-018-0176-5

**Published:** 2018-08-20

**Authors:** Bongkoch Turathum, Sittiruk Roytrakul, Chinarat Changsangfa, Morakot Sroyraya, Supita Tanasawet, Yindee Kitiyanant, Kulnasan Saikhun

**Affiliations:** 1 Department of Anatomy, Faculty of Science, Mahidol University, Bangkok, 10400 Thailand; 2 National Center for Genetic Engineering and Biotechnology, National Science and Technology Development Agency, Pathumthani, 12121 Thailand; 3 Institute of Molecular Biosciences, Mahidol University, Nakhon Pathom, 73170 Thailand; 4 Department of Anatomy, Faculty of Science, Prince of Songkla University, Hat Yai, 90110 Thailand

**Keywords:** Proteome, Cat oocyte, Cryopreservation, Vitrification

## Abstract

**Background:**

The domestic cat serves as an animal model for assisted reproductive studies of endangered felid species. To date, there are no data on the protein alterations following cryopreservation of oocytes in felid family.

**Methods:**

Immature (germinal vesicle) domestic cat oocytes were vitrified in the vitrification solution containing 35% ethylene glycol, 20% DMSO and 0.5 mM sucrose. The vitrified-warmed oocytes were matured (metaphase II) in vitro and subjected to proteomic analysis using 1DE SDS-PAGE prefractionation combined with LC–MS/MS.

**Results:**

A total of 1712 proteins were identified in in vitro matured oocytes. Of the 1712 proteins, 1454 proteins were found in both groups, whereas, 258 proteins were differentially expressed between control and vitrified-warmed groups. In vitrified-warmed oocytes, the missing proteins were membrane and nuclear proteins; whereas, apoptosis and DNA repair proteins were overrepresented.

**Conclusions:**

The identified missing and overexpressed proteins in vitrified-warmed oocytes represent potential markers of cryoinjuries and the developmental pathways of oocytes. The findings of differential expressed proteins may contribute to effective ways of proteome analysis of oocyte/embryo quality in order to assess safety of cryopreservation in felid species.

## Background

The domestic cat serves as an animal model for reproductive studies of endangered or non domestic species. To date, cryopreservation of oocytes is an essential part in reproductive biotechnology, and is an interesting procedure to conserve female gametes. Cryopreservation serves as an option to preserve reproduction capacity in aggressive cancer treatment that threatens the health of somatic and germ cells in the ovary [1–3].

Oocyte cryopreservation in mammalian species was first reported in mice [4], and subsequently in cattle [5, 6], goats [7] and humans [8]. Previous studies showed that oocytes submitted for vitrification or slow freezing induced subcellular alterations or disturbances in the intrinsic cellular homeostasis as compared to untreated controls. Cryopreservation may contribute to abnormal alignment of chromosomes in metaphase II (MII) oocytes, abnormal gene expression, increased reactive oxygen species, or high levels of DNA fragmentation [9–12]. Vitrification in human oocytes resulted in decreased overall mRNA abundance [13]. Oocytes exposed to high concentrations of cryoprotective agents during vitrification such as ethylene glycol (EG), dimethyl sulfoxide (DMSO) or propylene glycol (PG) might induce protein and subcellular alterations [14, 15], both of which could affect gene expression in either the oocyte or in the somatic cells of the follicle, and ultimately affect the oocyte proteome and developmental ability [16, 17]. Moreover, vitrification causes transient loss of communication between the oocyte and the surrounding granulosa cells [18], disrupting control of inner membrane potential in mitochondria [19]. These effects are in accordance with gene expression, paracrine signaling either by growth factors [20, 21] or follicular-fluid meiosis-activating substance [22], which ultimately induce alterations in the pattern of expression and the proteome of the oocyte.

Mass spectrometry is an integral technique used in proteomics, allowing the elucidation of the protein levels and compositions in cells. Protein profiles in oocytes and preimplantation embryos have been examined in mice and other species [3, 23–25]; however, there are no data on if and how the cryopreservation process affects proteins in felid family. Thus, the purpose of the present study is to identify the alterations of protein expression after cryopreservation of domestic cat oocytes.

## Methods

### Chemicals

All chemicals in this study were purchased from Sigma Chemical Company (Sigma, St. Louise, MO, USA), unless indicated otherwise. Media were prepared once per week, filtered (0.2 μm, Sartorius, Minisart, CA, USA) and kept in sterile bottles. Synthetic oviductal fluid (SOF) cultured media were incubated at 38.5 °C under 5% CO_2_ in air at least 4 h before use.

### Collection oocytes

Ovaries were obtained from healthy adult females of various breeds (> 6 months old) by ovariohysterectomy at the veterinary clinic of the Veterinary Public Health Division, Bangkok Metropolitan Administration. Ovaries were placed in 0.9% NaCl (containing 100 IU/mL penicillin) and transported to the laboratory (at 4 °C) within 2–4 h after removal. Ovaries were washed three times in 0.9% NaCl containing 100 IU/mL penicillin. To collect cumulus-oocyte complexes (COCs), ovaries were sliced repeatedly in Petri dishes containing TCM 199 (Invitrogen, Carlsbad, CA, USA) supplemented with 25 mM HEPES, 0.1% polyvinylalcohol, 0.1 mM glutamine, 2.5 mM sodium pyruvate and 1% penicillin streptomycin. COCs were washed and graded according to their investments and cytoplasm under a stereomicroscope. Grade 1 oocytes were surrounded by more than five layers of compacted cumulus cells and containing homogeneous dark cytoplasm. Grade 2 oocytes were surrounded by three to five layers of cumulus cells with homogeneous dark cytoplasm. Grade 3 oocytes were partially surrounded by cumulus cells and lacked homogenous cytoplasm. Grade 4 oocytes were without surrounding cumulus cells (denuded) and lacked homogenous cytoplasm. Only COCs with more than three layers of compacted cumulus cells and homogeneous dark cytoplasm (grades 1 and 2) were selected for the experiments.

### Vitrification and warming of immature oocytes

Cat oocytes were vitrified in straw (in-straw vitrification) using the procedures of Murakami et al. [26] with minor modifications. Immature oocytes (germinal vesicle stage, GV) were exposed to the holding medium (HM) (TCM 199 + 20 mM Hepes + 20% FBS) for 5 min, equilibrated in HM + 4% ethylene glycol for 3 min and then placed in a 5 μL drop of the vitrification solution (HM + 35% ethylene glycol + 20% DMSO + 0.5 mM sucrose) for 30 s. Groups of 10 oocytes were loaded into 0.25-mL plastic straws as followed: a 4-cm length straw was filled with HM containing 0.5 mM sucrose followed by a 0.5-cm air bubble, 0.5 cm of vitrification solution, an air bubble (about 0.5 cm), 1.0 cm of vitrification solution (about 20 µL) containing the oocytes, an air bubble (about 0.5 cm), vitrification solution (about 0.5 cm), and an air bubble (the remainder of the straw). The straws were sealed with heat. The straws were placed into liquid nitrogen (LN_2_) vapor (− 180 °C) for 3 min, then plunged into LN_2_ and stored at least 1 week before warming. The warming was performed by holding the straws in the air for 10 s and then plunging them into 37 °C water for 15–20 s. The contents of the straws were expelled into 37 °C of 0.3 M trehalose solution for 1 min, which rehydrated the oocytes. The oocytes were then washed and incubated in HM medium for 5 min before further evaluation.

### Viability assessment of vitrified-warmed oocytes

Vitrified warmed oocytes were assessed for viability based on their morphology as described previously [27]. COCs that were surrounded with compacted cumulus cells of symmetrical shape, with homogeneous cytoplasm, and showed no signs of lysis were classified as of normal morphology, thus, were subjected to in vitro maturation. In contrast, COCs with damaged cumulus cells, ruptured zona pellucida or leakage of cytoplasm were classified as abnormal oocytes, thus, were discarded.

### In vitro maturation

The COCs were cultured in Dulbecco’s Modified Eagle’s Medium (DMEM) supplemented with 1 µg/mL of follicle stimulating hormone (FSH), 1 µg/mL luteinizing hormone (LH) and 1 µg/mL estradiol. DMEM is a modification of Basal Medium Eagle (BME) that contains a four-fold higher concentration of amino acids and vitamins, as well as additional supplementary components. Ten COCs were cultured for 24 h in Petri dishes containing 100 μL/drop DMEM under mineral oil. The dishes were held in an incubator at 38.5 °C in a humidified atmosphere of 5% CO_2_. After culturing for 24 h, COCs were denuded of cumulus cells by exposure to 0.5% hyaluronidase for 5 min and gentle pipetting. The denuded oocytes were washed and characterized under a stereomicroscope for the MII stage by the presence of the first polar body within the perivitelline space. Oocytes with MII stage from control and vitrified-warmed groups were collected and prepared for protein fractionation by SDS-PAGE and analysis by LC–MS/MS.

### Protein extraction

A total of 1800 MII Oocytes derived from control and vitrified-warmed groups were washed three times in PBS. Lysis was carried out by adding 150 μL of 0.5% SDS supplemented with protease cocktail inhibitor to denuded oocytes, sonication in an ultrasonic bath for 20 min, 30 s vortexing, and 10 min incubation on ice. Lysate was centrifuged at 14,000 rpm, 4 °C for 20 min. The supernatant was collected and stored at − 80 °C.

### Determination of protein concentration by Lowry method

The pellets were resuspended in 0.5% SDS and protein concentration was measured using the Lowry method [28]. The absorbance at 750 nm (OD_750_) was measured and the protein concentration was calculated using the standard curve, plotted between OD_750_ on the Y-axis and BSA concentration (μg/mL) on the X-axis.

## 1D polyacrylamide SDS gel electrophoresis

Proteins were fractionated on SDS-PAGE mini slab gel [8 cm (L) × 9 cm (W) × 0.1 cm (H), Hoefer miniVE, Amersham Biosciences, UK]. The polyacrylamide gel was prepared according to the standard method described by Laemmli [29]. SDS gel electrophoresis was performed using a 4% stacking gel and a 12.5% separating gel. The protein lysate of 900 GV and MII oocytes were mixed with 5× sample buffer (0.125 M Tris–HCl pH 6.8, 20% glycerol, 5% SDS, 0.2 M DTT, 0.02% bromophenol blue) and heated at 95 °C for 10 min before loading onto the 12.5% SDS-PAGE. Electrophoresis was performed at a voltage of 70 V in SDS electrophoresis buffer (25 mM Tris–HCl pH 8.3, 192 mM glycine, 0.1% SDS). After the electrophoresis finished, gels were silver stained as described previously [30].

### Gel slicing and tryptic in-gel digestion

After silver staining, the bands in 12.5% gel were sliced into 15 pieces per lane. Each gel slice was cut into 1 mm pieces, and depending on the size of each slice, 70–90 pieces were digested by trypsin. The gel pieces were subjected to in-gel digestion using an in-house method developed by Proteomics Laboratory of the Genome Institute at the National Center for Genetic Engineering and Biotechnology (BIOTEC), Thailand. Briefly, the gel slices were dehydrated with 100% acetonitrile (ACN) three times, reduced with 10 mM DTT in 10 mM ammonium bicarbonate at room temperature for 1 h, and alkylated at room temperature for 1 h in the dark in the presence of 100 mM iodoacetamide (IAA) in 10 mM ammonium bicarbonate. After alkylation, the gel pieces were dehydrated twice with 100% ACN for 5 min. To perform in-gel digestion of proteins, 20 µL of trypsin solution (10 ng/µL trypsin in 50% ACN/10 mM ammonium bicarbonate) was added to the gels, followed by incubation at room temperature for 20 min. To keep the gels immersed throughout digestion, 30 µL of 30% ACN was added and incubated at 37 °C overnight. To extract peptide digestion products, 30 µL of 50% ACN in 0.1% formic acid was added into the gels and incubated at room temperature for 10 min in a shaker, and repeated three times. Extracted peptides were then collected, dried by vacuum centrifuge, and kept at − 80 °C for further mass spectrometric analysis. Prior to LC–MS/MS analysis the peptides were dissolved in 20 µL 0.1% formic acid.

### HCTUltra LC–MS analysis

Peptide solutions were analyzed using an HCTultra PTM Discovery System (Bruker Daltonics Ltd., U.K.) coupled with an UltiMate 3000 LC System (Dionex Ltd., U.K.). Peptides were separated on a nanocolumn (PepSwift monolithic column 100 µm i.d. × 50 mm). Eluent A was 0.1% formic acid and eluent B was 80% acetonitrile in water containing 0.1% formic acid. Peptide separation was achieved with a linear gradient from 10% to 70% B for 13 min at a flow rate of 300 nL/min, including a regeneration step at 90% B and an equilibration step at 10% B. Each run took 20 min. Peptide fragment mass spectra were acquired in data-dependent AutoMS (2) mode with a scan range of 300–1500 m/z, 3 averages, and up to 5 precursor ions selected from the MS scan 50–3000 m/z.

### Protein quantitation and identification

For protein quantitation, DeCyder MS Differential Analysis software (DeCyderMS, GE Healthcare) [31] was used. Acquired LC–MS raw data were converted and the PepDetect module was used for automated peptide detection, charge state assignments, and quantitation based on the peptide ions signal intensities in MS mode. The analyzed MS/MS data from DeCyderMS were submitted to a database search using the Mascot software (Matrix Science, London, UK) [32]. The data was searched against the NCBI database for protein identification. Database interrogation was; taxonomy (Viridiplantae); enzyme (trypsin); variable modifications (carbamidomethyl, oxidation of methionine residues); mass values (monoisotopic); protein mass (unrestricted); peptide mass tolerance (1 Da); fragment mass tolerance (± 0.4 Da), peptide charge state (1 +, 2 + and 3 +), max missed cleavages (1) and instrument = ESI − TRAP. Proteins with at least one peptide with an individual mascot score corresponding to *P *< 0.05 were considered to be identified proteins.

Data normalization and quantification of the changes in protein abundance between the control and treated samples were performed and visualized using MultiExperiment Viewer (Mev) software version 4.6.1 [33]. Briefly, peptide intensities from the LC–MS analyses were transformed and normalized using a mean central tendency procedure. Using the software, we performed analysis of variance (ANOVA) tests to identify proteins with statistical differences (*P* < 0.05) between treatment groups.

Gene ontology annotation, including molecular function and biological process, was assigned to each identified protein according to the Uniprot database (http://www.uniprot.org). The identified proteins were also submitted to The Search Tool for the Retrieval of Interacting Genes (STRING) (http://string-db.org) to identify cellular functions and annotate all functional interactions among proteins in the cell [34].

## Results

### Viability assessment of vitrified-warmed oocytes

**Fig. 1:**
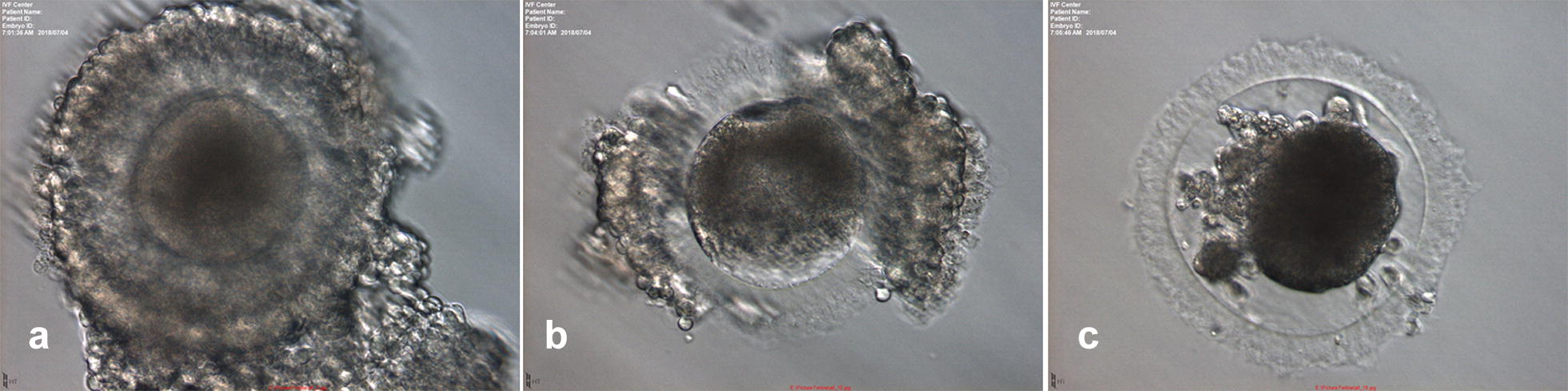
Morphology of vitrified-warmed oocytes after warming (×200). **a** Normal oocyte with compacted cumulus cells, symmetrical shape and no signs of lysis. **b** Abnormal oocyte with damaged cumulus cells. **c** Abnormal oocyte with ruptured zona pellucida and leakage of cytoplasm

A total of 1460 oocytes were vitrified. After warming, the oocytes were evaluated for the morphology under stereomicroscope. We classified normal oocytes as having compact cumulus cells, symmetrical shape, and no signs of lyses (Fig. 1a). Abnormal oocytes, identified as having damaged cumulus cells (Fig. 1b) and ruptured zona pellucida or leakage of cytoplasm (Fig. 1c) were excluded. The percentage of normal vitrified-warmed oocytes after warming was 95.7%.

### In vitro maturation of oocytes

To investigate protein changes during IVM of oocytes following vitrification, immature oocytes from control (fresh oocytes) and vitrified-warmed groups were cultured for IVM. Following 24 h of IVM, the maturation rate was 54.04% for the control group and 21.58% for the vitrified-warmed group.

### Quantitative proteome profile in control and vitrified-warmed MII oocytes

**Fig. 2:**
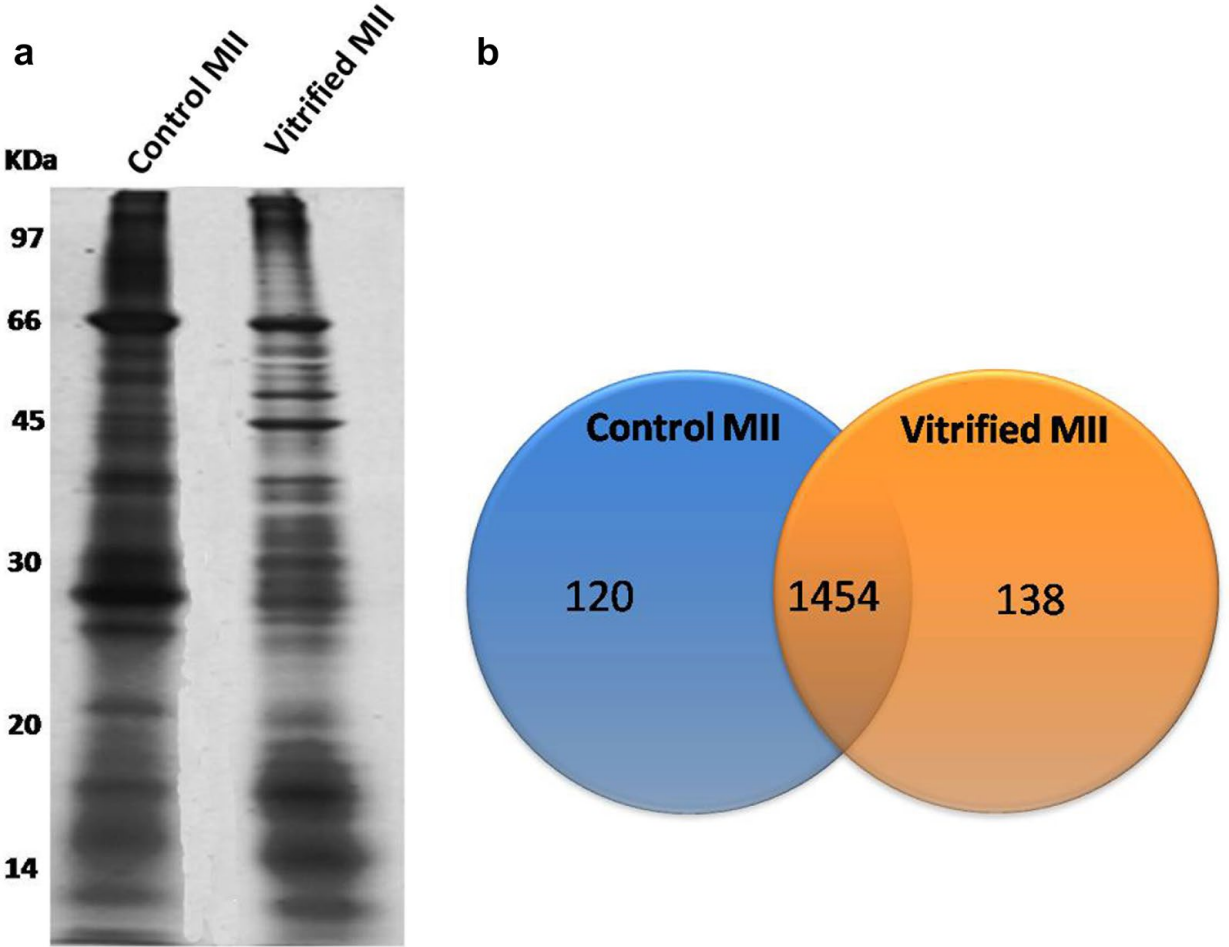
SDS-PAGE fractionation of MII oocytes derived from control and vitrified-warmed groups (**a**). Twenty micrograms of protein from each group were fractionated on a 12.5% acrylamide gel (8 × 13 cm). Pooled protein was run on the gel and subsequently stained with silver staining. Each gel lane was sliced and then subjected to in-gel digestion prior to LC/MS–MS analysis. Lane 1: total protein of MII oocytes in control group and Lane 2: total protein of MII oocytes in the vitrified-warmed group. Venn diagram of identified proteins from MII oocytes derived from control and vitrified-warmed groups (**b**)

SDS-PAGE protein separation with protein identification by LC–MS/MS was used as a classified proteomic approach to investigate protein expression changes of oocytes in control and vitrified-warmed groups during IVM. Proteins extracted from MII oocytes in both group were resolved by SDS-PAGE on 12.5% polyacrylamide gel. A representative SDS-PAGE image is shown in Fig. 2a. Gels were sliced into 15 pieces and subjected to in-gel tryptic digestion. Peptides were analyzed by LC–MS/MS. A total of 1712 protein identifications were made from 20 μg of protein. Of these 1712 proteins, 1454 proteins were found in both groups and 258 proteins were differentially expressed between control and vitrified-warmed groups (Fig. 2b).

**Fig. 3:**
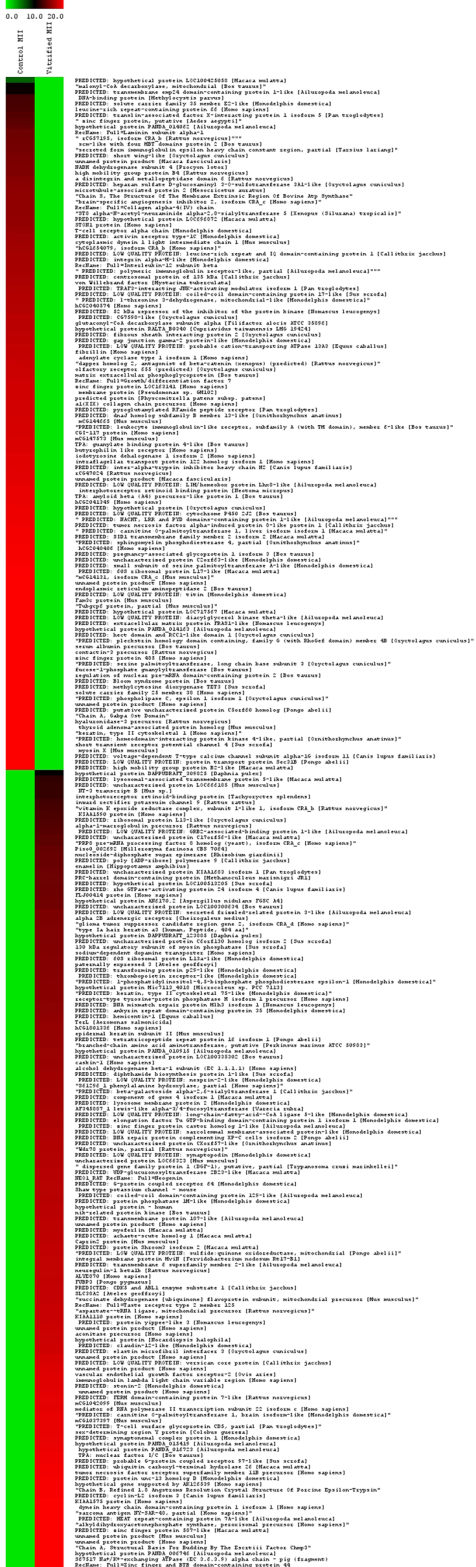
Time-profile expression pattern of 258 differentially expression proteins in MII oocytes derived from control and vitrified-warmed groups. An over expression pattern of 258 proteins was performed with MultiExperiment Viewer (MeV, version 4.9.0) software. All quantitative information was marked using a color scale ranging from green, black, and red for no changes, a little, and the highest up-regulation ratio, respectively

A total of 258 proteins showed expression difference between control and vitrified-warmed MII oocytes after IVM. A total of 74 proteins were identified from 258 differentially expressed proteins based on at least two unique tryptic peptides with protein ID scores > 10 and increased expression at least two times across time points. The alteration of proteins in MII-stage oocytes of the control group compared to the vitrified-warmed group were shown using MultiExperiment Viewer (MeV, version 4.9.0) software (Fig. 3). In vitrified-warmed MII oocytes, 32 proteins were not expressed and 42 proteins were over-expressed compared to the control group.

### Distribution of biological function and cellular components of miss-expressed proteins

**Fig. 4:**
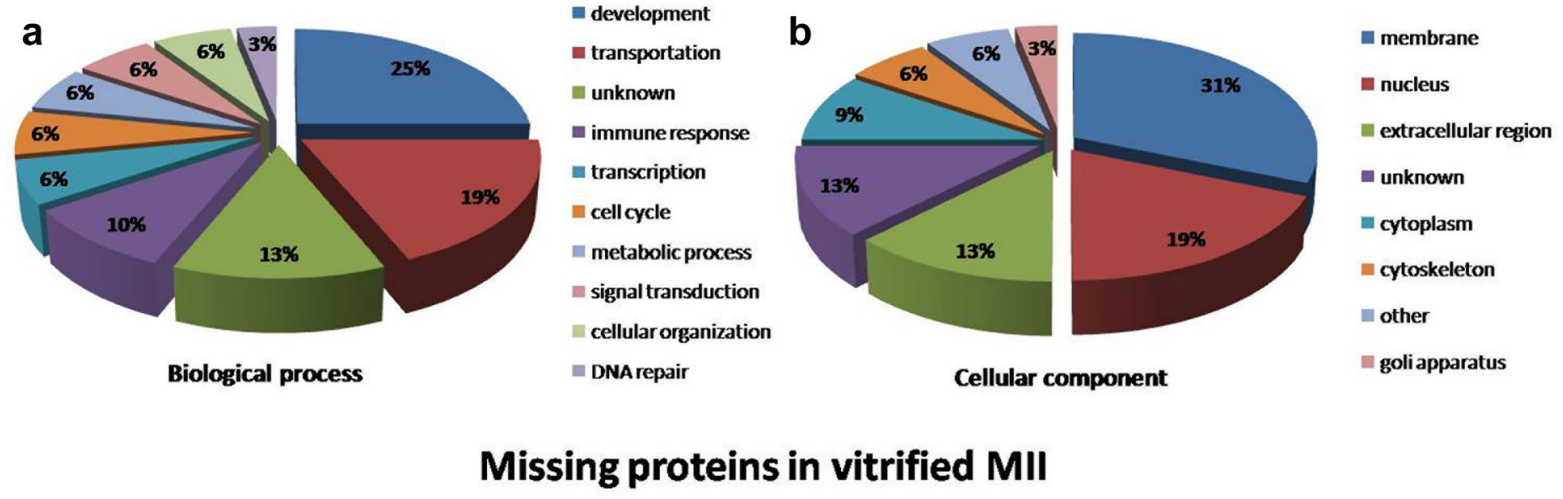
Pie charts of the biological processes (**a**) and cellular components (**b**) of missing proteins in vitrified-warmed MII oocytes after IVM. The biological processes and cellular compartments were assigned to the protein identified according to the Uniprot database (http://www.uniprot.org) and gene ontology (GO)

The 32 miss-expressed proteins were classified according to their biological function (Fig. 4). The assigned functions of miss-expressed proteins were development (25%), transportation (19%), immune response (10%), metabolic process (6%), cell cycle (6%), cellular organization (6%), transcription (6%), signal transduction (6%), DNA repair (3%), and unknown function (13%). Details of the miss-expressed proteins and their biological function are presented in Table 1.

**Table 1:** An example of missing proteins in vitrified-warmed MII oocytes following IVM are listed based on their involvement in biological processes and protein scores

Protein name	Biological process	ID Score	Database	Species	Peptide sequence
Growth/differentiation factor 7	Development	25.64	gi|50400625		SPGGGGGGGR
Contactin-3 precursor	Development	20.40	gi|9506949	*Rattus norvegicus*	SDGMPFPTKIK
Hyaluronidase-3 precursor	Development	19.98	gi|67514570	*Rattus norvegicus*	LPPAYHQTFVR
Fam3c protein	Development	15.59	gi|47125071	*Mus musculus*	SARSGGGGGGGTSR
Microtubule-associated protein 2 (MAP2)	Development	15.22	gi|291508792	*Mesocricetus auratus*	ATELKFEMSPELALSSK
a1(XIX) collagen chain precursor	Development	12.78	gi|624871	*Homo sapiens*	TGAQGPAGEPGIQGPR
Brain-specific angiogenesis inhibitor 2 (BAI2)	Development	12.48	gi|119627996	*Homo sapiens*	MENTGWMGK
Titin	Development	10	gi|334329989	*Monodelphis domestica*	ELTPGPKYK
Voltage-dependent T-type calcium channel subunit alpha-1G isoform 11	Transportation	15.00	gi|73966393	*Canis lupus* familiaris	HGGSLER
Myosin X	Transportation	12.67	gi|6996558	*Mus musculus*	SMILR
Gap junction gamma-2 protein-like	Transportation	12.34	gi|334324640	*Monodelphis domestica*	SQQEKSSR
Transmembrane emp24 domain-containing protein 1-like	Transportation	11.88	gi|301771990	*Ailuropoda melanoleuca*	ESIETMKTR
STON1 protein	Transportation	11.14	gi|111309209	*Homo sapiens*	MFSSRNK
Integrin alpha-M-like	Immune response	13.43	gi|126334040	*Monodelphis domestica*	THTASGIR
Tumor necrosis factor alpha-induced protein 8	Immune response	11.63	gi|296232583	*Callithrix jacchus*	ICEGLGRMLDDGSL
Interleukin-12 subunit beta	Immune response	10.53	gi|81914499		NSEAVGSGK
Iodotyrosine dehalogenase 1 isoform 2	Metabolic process	24.17	gi|42794271	*Homo sapiens*	EATVPDLK
Inter-alpha-trypsin inhibitor heavy chain H2	Metabolic process	12.08	gi|73949158	*Canis lupus* familiaris	TTGLVR
Polymeric immunoglobulin receptor-like, partial	Signal transduction	21.59	gi|301767800	*Ailuropoda melanoleuca*	SQIISVTLNPVSK
52 kDa repressor of the inhibitor of the protein kinase	Signal transduction	13.49	gi|332210861	*Nomascus leucogenys*	FNTSEGHHADMYR
TPA: amyloid beta (A4) precursor-like protein 1	Transcription, cell cycle	11.65	gi|296477814	*Bos Taurus*	SWPPGGR
Zinc finger protein	Transcription	12.83	gi|157105094	*Aedes aegypti*	HMQAAHGK
Chain A, Gabpa Ost Domain	Transcription	10.10	gi|178847068		MAECVSQAIDINEPIGNLK
Butyrophilin like receptor	Cellular organization	10.59	gi|4587209	*Homo sapiens*	HGQAELR
Gamma-tubulin complex component 6 (Tubgcp6)	Cellular organization	10	gi|35505467	*Mus musculus*	GPGKSR

Only protein with proteins ID score > 10 are listed

The miss-expressed proteins identified in terms of cellular components were assigned to membrane proteins (31%), nuclear proteins (19%), extracellular proteins (13%), cytoplasmic proteins (9%), cytoskeletal proteins (6%), the golgi apparatus (3%), localization in other region (6%), and unknown localization (13%).

### Distribution of biological function and cellular components of over-expressed proteins

**Fig. 5:**
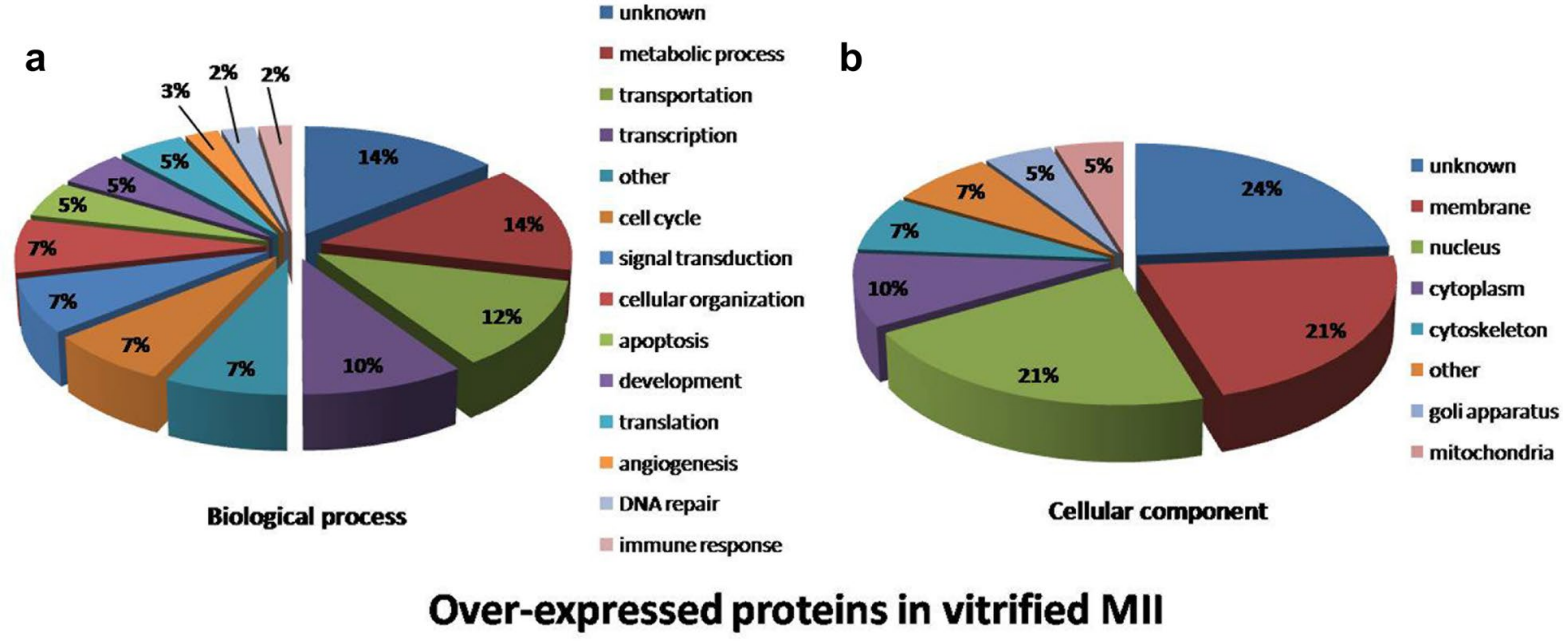
Pie charts of biological processes (**a**) and cellular components (**b**) of an over-expressed proteins in vitrified-warmed MII oocytes after IVM. The biological processes and cellular compartments were assigned to the protein identified according to the Uniprot database (http://www.uniprot.org) and gene ontology (GO)

The 42 over-expressed proteins were classified according to their biological functions (Fig. 5). The assigned functions of over-expressed proteins were metabolic process (14%), transportation (12%), transcription (10%), cell cycle (7%), signal transduction (7%), cellular organization (7%), others (7%), apoptosis (5%), development (5%), translation (5%), angiogenesis (3%), DNA repair (2%), immune response (2%), and unknown function (14%). Details of the over-expressed proteins and their biological function are presented in Table 2.

**Table 2:** An example of over-expressed proteins in vitrified-warmed MII oocytes following IVM are listed based on their involvement in biological processes and protein scores

Protein name	Biological process	ID Score	Database	Species	Peptide sequence
Alkyldihydroxyacetonephosphate synthase, peroxisomal precursor	Metabolic process	15.49	gi|4501993	*Homo sapiens*	RYPLSGMGLPTFK
ALYE870 (carbohydrate Sulfotransferase 5)	Metabolic process	13.24	gi|37182346	*Homo sapiens*	AADAPFVINAIIR
Sulfide:quinone oxidoreductase, mitochondrial	Metabolic process	12.99	gi|297696565	*Pongo abelii*	GYWGGPAVLR
Carnitine O-palmitoyltransferase 1	Metabolic process	11.08	gi|334329080	*Monodelphis domestica*	AGNAVYAMMR
Vitamin K epoxide reductase complex	Metabolic process	10	gi|149063156	*Rattus norvegicus*	QLNSDFIK
Stonin-2	Transportation	15.92	gi|126282180	*Monodelphis domestica*	DEFSGVLR
Inward rectifier potassium channel 9	Transportation	11.70	gi|609672	*Rattus rattus*	TSPAPR
Shaw type potassium channel—mouse	Transportation	11.63	gi|2143521		HGGGGGDSGK
Lysosomal-associated transmembrane protein 5-like	Transportation	11.48	gi|297282785	*Macaca mulatta*	GGDSSTMDPR
Unnamed protein product	Transportation	10.80	gi|16551721	*Homo sapiens*	GIGGHLDSLLGR
Diphthamide biosynthesis protein 1-like	Translation	9.16	gi|335298294	*Sus scrofa*	ERPLQAAGR
Sex-determining region Y protein	Transcription	20.92	gi|323530757	*Colobus guereza*	KMELDNR
FUBP3	Transcription	13.76	gi|124054230	*Pongo pygmaeus*	XAREMVLEIIR
Zinc finger protein castor homolog 1-like	Transcription	10.11	gi|301784136	*Ailuropoda melanoleuca*	DDSGPGAK
KIAA1575 protein	Transcription	9.06	gi|27529915	*Homo sapiens*	DVTDKPLDLSSK
Synaptonemal complex protein 1	Cell cycle	19.87	gi|334324527	*Monodelphis domestica*	DELDAVK
CDK5 and ABL1 enzyme–substrate 1	Cell cycle	15.35	gi|296222383	*Callithrix jacchus*	DSTQAGDLK
Sarcoma antigen NY-SAR-48, partial	Cell cycle	11.35	gi|29164881	*Homo sapiens*	GKMADSSGR
Unnamed protein product	Signal transduction	14.55	gi|34531434	*Homo sapiens*	LLLETGMK
Probable G-protein coupled receptor 97-like	Signal transduction	11.92	gi|311257217	*Sus scrofa*	GSSGDWSSK
Nik-related protein kinase	Signal transduction	10.19	gi|300793971	*Bos Taurus*	AMEVRSSQSR
Dynein heavy chain domain-containing protein 1 isoform 1	Cellular organization	24.99	gi|222144249	*Homo sapiens*	GGPIK
Protein Shroom3 isoform 2	Cellular organization	18.02	gi|297292672	*Macaca mulatta*	SSSMDNTSAR
Transmembrane protein 187-like	Cellular organization	14.76	gi|301786893	*Ailuropoda melanoleuca*	LSPEGKVH
KIAA1110 protein	Cellular organization	11.79	gi|5689557	*Homo sapiens*	GVDDGADIPR
Protein yippee-like 3	Apoptosis	14.59	gi|332265954	*Nomascus leucogenys*	ALGVPR
Receptor-type tyrosine-protein phosphatase H	Apoptosis	14.26	gi|241896924	*Homo sapiens*	NATTAPNPVR
Wdr78 protein, partial	Development	18.30	gi|53734209	*Rattus norvegicus*	VTEDEVK
Thrombopoietin receptor-like	Development	15.63	gi|126305853	*Monodelphis domestica*	NKPPP

Only protein with proteins ID score > 10 and increased expression at least two times across time points are listed

The cellular components of over-expressed proteins were assigned to membrane proteins (21%), nuclear proteins (21%), cytoplasmic proteins (10%), cytoskeletal proteins (7%), golgi apparatus (5%), mitochondrial proteins (5%), localization in other regions (7%) and unknown localization (24%).

## Discussion

It is well accepted that cryopreservation is an essential technique to preserve oocytes in cats and other mammalian species. In cats, cryo-devices, cryoprotectants and vitrification procedures affected viability, nuclear maturation and subsequent embryonic development of vitrified-warmed oocytes [35, 36]. The method of in-straw vitrification used in our study was successfully used for cryopreservation of in vitro matured cat oocytes [26]. In the present study, the rates of normal oocytes after warming and maturation after IVM was 95.7 and 21.58%, respectively. Immature cat oocytes vitrified in-straw resulting in a morphologically normal after warming (75.0%) but only 37.5% of these oocytes resumed meiosis and developed to the MII stage [27]. Vitrification of immature cat oocytes using open pulled straws (OPS) significantly decreased the rates of cleavage (18.6% vs 48.2%) and blastocyst development (4.3% vs 20.6%) as compared to the control [37]. It has been reported that slow freezing represents a suitable method of GV stage cat oocytes banking since it more efficiently preserves the functional coupling with cumulus cells after thawing as well as nuclear and cytoplasmic competence [38]. Recently, Fernandez-Gonzalez and Jewgenow [39] demonstrated that immature cat COCs can be vitrified either by IZW method or by commercial Kitazato^®^ kit. Vitrified-warmed oocytes obtained from both vitrification methods were able to undergo maturation, fertilization and subsequent development to the morula stage. However, the use of slush nitrogen (SN2) to increase cooling rates is not a benefit for these procedures. Although immature cat oocytes were successfully cryopreserved either by vitrification or slow freezing, however, to improve rate of maturation and further fertilization after cryopreservation of immature oocytes, we need to study the effect of cryoinjuries, understand molecular mechanisms and proteins that involved in meiotic maturation.

The present study used proteomic analysis to determine the effect of a cryopreservation procedure on differential protein profiles in feline oocyte during meiotic maturation. This is the first report indicating that 258 proteins were differentially expressed in MII-stage oocytes by comparing control and vitrified-warmed groups. Moreover, a total of 74 proteins were identified from 258 differentially expressed proteins. These results suggested that the feline IVM process might depend upon 74 specific proteins that are differentially expressed. These proteins might have important roles in the molecular events involving feline oocyte development. The identified missing and overexpressed proteins in vitrified-warmed oocytes may be associated with the reduction of oocytes resumed meiosis and reached to the MII stage. Thirty-two proteins were identified as miss-expressed at least two times across time points. Biological function of the major group of miss-expressed proteins was developmental, although transportation proteins were also highly represented.

Proteins involved in development were the most frequently over-expressed in vitrified-warmed MII oocytes. On the basis of the protein score, growth/differentiation factor 7 (GDF7) appeared to be one of development proteins identified; however, other over-expressed proteins involved in the development were also identified. These included the Contactin-3 precursor (CNTN3), Hyaluronidase-3 precursor (HYAL3), Microtubule-associated protein 2 (MAP2), FAM3C, a1(XIX) collagen chain precursor, BAI2 and Titin.

MAP2, the microtubule-associated protein family (MAPs) serves to stabilize microtubule (MT) growth by cross-linking MT with intermediate filaments and other MTs [40]. The expression of MAP2 is predominantly neuronal and more specifically in dendrites. Multiple isoforms of MAP2 exist in human cells, with high molecular weight isoforms MAP2a and MAP2b, and low molecular weight isoforms MAP2c and MAP2d being expressed in different stages of development. MAP2a and MAP2b are concentrated in dendrites of neural cells, with MAP2a being expressed at the late stages of development and MAP2b being expressed at both embryonic and adult stages [41]. BAI2 is a member of the G protein couple receptor (adhesion GPCR) family, which play a role in angiogenesis [42]. HYAL3, a mammalian hyaluronidases, is ubiquitously expressed. The absence of HYAL3 was shown to increase the apoptotic granulosa cells and the atretic follicles [43]. GDF7 is a growth/differentiation factor (GDFs). GDFs are a subgroup of the bone morphogenetic proteins (BMPs), which are well known for their role in joint formation and chrondrogenesis. In the absence of GDF7, the rate of endochondral bone growth was affected by modulation of hypertrophic phase duration in the growth plate chondrocytes. GDF7 has a role in long bone mechanics, tendon maintenance and repair, neural tissue modulation and maintenance, dental development and reproductive organ formation.

Titin is a calcium-responsive protein, which is a group that is responsible for oocyte meiosis resumption [44]. Expression of titin in the human trophoblast is involved in the processes of placentation and embryo development [45]. Contactins, a subgroup of molecules belonging to the immunoglobulin superfamily, consists of six members: contactin, TAG-1, BIG-1, BIG-2, NB-2 and NB-3. BIG-1 is expressed uniquely in a group of neurons, such as Purkinje cells of the cerebellum, granule cells of the hippocampal dentate gyrus, and the superficial layer neurons of the cerebral cortex [46]. Type XIX collagen is an extracellular matrix protein that acts as a cross-bridge between fibrils and other extracellular matrix molecules. Collagen XIX is distributed in vascular, neuronal, mesenchymal, and some epithelial basement membrane zones [47]. Collagen XIX is expressed by central neurons and is necessary for the formation of hippocampal synapses [48], the assembly of embryonic matrices, and the maintenance of specific adult tissues [49]. FAM3C is a protein that is involved in the epithelial to mesenchymal transition. It is expressed in the ganglion cells of the retina and is involved in retinal laminar formation processes in vertebrates [50].

Miss-expressed proteins that involved in transportation include the gap junction gamma-2 protein (GJC2), STON1, transmembrane emp24 domain-containing protein 1 (TMED1), voltage-dependent T-type calcium channel, myosin X (MYO10), and the extracellular matrix protein FRAS1. GJC2 also known as connexin-46.  (Cx46.6) and connexin-47 (Cx47) and gap junction alpha-12 (GJA12), plays a key role in central myelination and is involved in peripheral myelination in humans [51]. Myosin X, a member of the myosin superfamily, is ubiquitously expressed in various mammalian tissues. MYO10 binds to actin filaments and actin bundles, its tail domain binds to integrins, and it mediates cargo transport along actin filaments. MYO10 appears to play a role in cytokinesis, cell shape, and cell adhesion, as well as stimulating the neurite outgrowth and axon guidance, formation of the podosome belt in osteoclasts [52], and integration of F-actin and microtubule cytoskeletons during meiosis [53].

During vesicular transport between the endoplasmic reticulum and the Golgi, members of the transmembrane emp24 domain (TMED) protein family form heterooligomeric complexes that facilitate protein cargo transportation and secretion. TMED1 is an important player in innate immune signaling [54]. Extracellular matrix protein FRAS1 is an extracellular matrix protein that appears to function in the regulation of epidermal-basement membrane adhesion and organogenesis during development. Lack of FRAS1 causes Fraser syndrome, a multisystem malformation that can include craniofacial, urogenital, and respiratory system abnormalities [55].

Voltage-gated Ca^2+^ channels are key players for regulating Ca^2+^ influx in cells and have been described in single and two-cell mouse embryos [56]. Proteins involved in the cell cycle, signal transduction, and transcription were also mis-expressed in post-thawed MII oocytes. Missing proteins involved in the cell cycle are activin receptor type-1C and amyloid beta (A4) precursor. Activin receptor type-1C (ACVR1C) was found in several types of cells including granulose cells, cumulus cells, and oocytes. Activin produced by granulosa cells, and acts both on the oocyte and the granulosa cells through activin receptor 2 types; Type I and II [57]. Activin promotes the in vitro maturation and fertilization in primate oocytes [58]. The amyloid-beta precursor protein (AbPP), a ubiquitously expressed adhesion and neuritogenic protein, is regulated by reproductive hormones including the gonadotropin luteinizing hormone (LH) in human neuroblastoma cells. AbPP also has structural similarity with growth factors and is expressed in human embryonic stem cells. An important function of this protein is its role in early human embryogenesis prior to the formation of neural precursor cells [59].

Missing proteins involved in transcription were Zinc finger and Chain A, Gabpα ost domain. A zinc finger is a small, functional, independently folded domain that coordinates one or more zinc ion to stabilize its structure through cysteine and/or histidine residues. Zinc finger proteins are expressed especially in mature oocytes and function in oocyte maturation and early embryogenesis. OMA1 and OMA2 are positive regulators of prophase progression during meiotic maturation in *C. elegant* [60]. When the function of Zinc finger protein (Zfp36l2) is disrupted, the embryos did not progress beyond the two-cell stage of development in mice [61]. GA-binding protein transcription factor subunits function as DNA-binding subunits. These proteins are likely involved in activation of cytochrome oxidase expression and nuclear control of mitochondrial function. GABPα plays a role in mitochondrial biogenesis. In mouse embryonic fibroblast, loss of Gabpα reduced mitochondrial mass, ATP production, oxygen consumption, and mitochondrial protein synthesis [62].

The polymeric immunoglobulin receptor (pIgR) and 52 kDa repressor of the inhibitor (P52rIPK) were involved in signal transduction of missing proteins. pIgR facilitates the secretion of the soluble polymeric isoforms of immunoglobulin A and immunoglobulin M. pIgR is widely present in all ectoderm- and endoderm-derived structures and was present in 4-week-old embryos [63]. P52rIPK is an upstream regulator of interferon-induced serine/threonine protein kinase R (PKR). This protein may block the PKR-inhibitory function of P58IPK, resulting in restoration of kinase activity and suppression of cell growth. P52rIPK is a novel protein kinase-regulatory system that encompasses an intersection of interferon-, stress-, and growth-regulatory pathways [64].

## Conclusions

The present study demonstrates the effect of cryopreservation on the alterations of protein expression in domestic cat oocytes using proteomic analysis. This is the first report indicating that 258 proteins were differentially expressed between vitrified-warmed and control groups. The identified missing and overexpressed proteins in vitrified-warmed oocytes associated with the reduction of oocytes resumed meiosis and reached to the MII stage indicating the suitable cryopreservation protocol needs to be established in felid species. Our findings provide important knowledge to better understand the molecular changes in cryopreserved oocytes and also establish the foundations of proteomic research aimed at improving the performance of oocytes cryopreservation in the domestic cat. Further experiments are required to investigate the functions of proteins expressed specifically in oocytes following vitrification to better understand the molecular mechanism of oocyte developmental pathways and ultimately improve the efficiency oocyte cryopreservation in felid species.
